# Arthroscopic anterior inferior iliac spine decompression does not alter postoperative muscle strength

**DOI:** 10.1007/s00167-018-5026-z

**Published:** 2018-06-28

**Authors:** Satoshi Tateishi, Yasuo Onishi, Hitoshi Suzuki, Makoto Takahashi, Junichiro Shiraishi, Christopher M. Larson, Soshi Uchida

**Affiliations:** 1 Department of Rehabilitation Medicine, Wakamatsu Hospital for the University of Occupational and Environmental Health, 1-17-1, Hamamachi, Wakamatsu, Kitakyushu, Fukuoka 808-0024 Japan; 2 Department of Orthopaedic Surgery, Faculty of Medicine, Wakamatsu Hospital for the University of Occupational and Environmental Health, 1-17-1, Hamamachi, Wakamatsu, Kitakyushu, Fukuoka 808-0024 Japan; 3 Minnesota Orthopedic Sports Medicine Institute at Twin Cities Orthopedics, 4010 West 65th Street, Edina, MN 55435 USA

**Keywords:** Subspinal impingement, Hip arthroscopy, Femoroacetabular impingement, Rehabilitation, Muscle strength, Anterior inferior iliac spine

## Abstract

**Purpose:**

The purpose of this study was to assess the additional effect of anterior inferior iliac spine (AIIS) decompression on knee extensor and hip flexor strength and compare functional outcomes after arthroscopic FAI correction with and without AIIS decompression.

**Methods:**

Sixty patients who underwent arthroscopic FAI correction surgery were divided into two groups matched for AIIS morphology: 31 patients who underwent arthroscopic FAI surgery only (without AIIS decompression) (FAI group) (AIIS Type I; *n* = 5, Type II; *n* = 26, Type III; *n* = 0) and 29 patients who underwent arthroscopic FAI surgery with AIIS decompression (AIIS group) (AIIS Type I; *n* = 5, Type II; *n* = 24, Type III; *n* = 0). Knee extensor and hip flexor strength were evaluated preoperatively and at 6 months after surgery. Patient-reported outcome (PRO) scores using the modified Harris hip score (MHHS), the nonarthritic hip score (NAHS) and iHOT-12 were obtained preoperatively and at 6 months after surgery.

**Results:**

In the AIIS group, there was no significant difference between knee extensor strength pre- and postoperatively (n.s.). In the AIIS group, hip flexor strength was significantly improved postoperatively compared to preoperative measures (*p* < 0.05). In the FAI group, there were no significant improvements regarding muscle strength (n.s.). While there were no significant differences of preoperative and postoperative MHHS and NAHS between both groups (MHHS; n.s., NAHS; n.s.), the mean postoperative iHOT-12 in the FAI group was inferior to that in the AIIS group. (*p* < 0.01). The revision surgery rate for the AIIS group was significantly lower compared with that in the FAI group (*p* < 0.05).

**Conclusion:**

Anterior inferior iliac spine decompression, as a part of an arthroscopic FAI corrective procedure, had a lower revision surgery rate and did not compromise knee extensor and hip flexor strength, and it improved clinical outcomes comparable to FAI correction without AIIS decompression. AIIS decompression for FAI correction improved postoperative PRO scores without altering the muscle strength of hip flexor and knee extensor.

**Level of evidence:**

III.

## Introduction

Femoroacetabular impingement (FAI) is one of the most common sources of hip pain and discomfort [9]. FAI is the most frequent indication for arthroscopic hip surgery. More recently, it has been increasingly recognized that there are extra-articular patterns of impingement such as ischiofemoral impingement and anterior inferior iliac spine (AIIS) impingement [8, 13, 15, 20, 26].

The AIIS represents the origin of the direct head of the rectus femoris tendon, and its bony morphology can be quite variable and result in impingement against the distal femoral neck with excessive distal and/or anterior extension [20]. Recently, it has been better defined that residual AIIS impingement is also one of the most common predictors of revision and poor clinical outcomes following arthroscopic FAI correction [19, 29]. Additionally, AIIS decompression can affect postoperative results following arthroscopic FAI surgery [19, 29, 30]. Therefore, the current authors perform an additional AIIS decompression as a part of an arthroscopic FAI corrective surgery if AIIS impingement is suspected based on examination, imaging, and intraoperative findings.

Hapa et al. described that AIIS decompression could be performed to minimize the invasion of the origin of the rectus femoris [12]. AIIS decompression was carefully performed to avoid invasion to the attachment of the rectus femoris. However, the current literature lacks a clear understanding of the effect of AIIS decompression on hip flexor and knee extensor strength. The purposes of this study were to examine the effect of additional AIIS decompression as part of an arthroscopic FAI correction on hip flexor and knee extensor strength as measured with a dynamometer. In addition, patient-related outcome measures were evaluated for patients after FAI correction with and without AIIS decompression, and the revision surgery rate was also examined. This study mainly focuses on investigating whether AIIS decompression as a part of FAI correction surgery affect hip flexor and knee extensor muscle strength or not. It was hypothesized that AIIS decompression does not affect hip flexion and knee extension muscle strengths, improves the clinical outcome measures and decreases the revision surgery rate.

## Materials and methods

Five hundred and fifty-four patients who underwent arthroscopic FAI corrective surgery by the primary author (SU) from August 2011 to July 2015 were retrospectively researched at our hospital.

An anterior impingement test was done with the patient supine, and the hip was rotated internally as it was flexed passively to approximately 90° and adducted [27]. If the patient complained of groin pain, this test was defined as positive. Radiographic evidence of a cam deformity was defined as alpha angle > 55° or head–neck offset < 8 mm on at least one radiographic view or computed tomography CT or magnetic resonance imaging MRI [7, 24]. Radiographic evidence of a pincer deformity was defined as a positive crossover sign in the presence of a lateral center edge angle (LCEA) ≥ 30°, a lateral CE angle (LCEA) of 40° and/or an acetabular inclination of < 0°, and it was also a positive sign of an ischial spine [18, 23, 34]. A radiographic FAI subtype was additionally classified as an isolated cam, isolated pincer, or combined FAI.

**Fig. 1:**
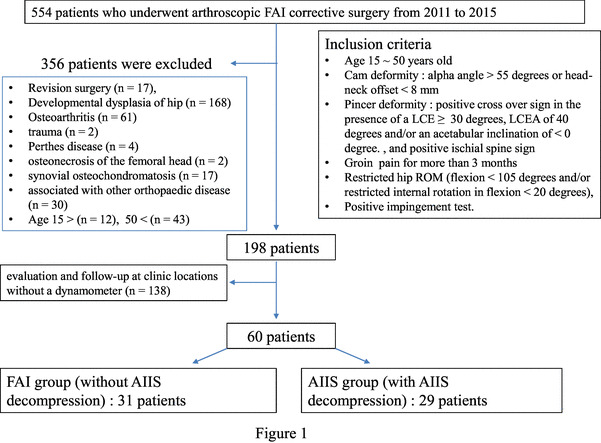
Flow chart of patient selection

Finally, 60 patients (34 male, 26 female) with a mean age of 29.6 ± 12.9 years (range 15–50) were enrolled in this study and evaluated preoperatively and postoperatively at a mean follow-up period of 181 ± 32 days. (Fig. 1).

AIIS morphology was assessed by three-dimensional computed tomography (3D-CT) and classified according to the Hetsroni et al. classification based on the relationship between the AIIS and the acetabular rim [14]. Type I is present when there is a smooth ilium wall between the AIIS and the acetabular rim; Type II is present when the AIIS extends to the level of the rim; and Type III is present when the AIIS extends distal to the acetabular rim [14]. The AIIS type was classified in the same method.

The current study consists of two non-randomized consecutive groups. Until March 2013, arthroscopic FAI correction and labral preservation (repair and/or reconstruction) was performed without AIIS decompression because of unawareness of the entity of subspine/AIIS impingement at the primary author’s institution. Thirty-one patients who underwent arthroscopic FAI surgery without AIIS decompression (Type I; *n* = 5, Type II; *n* = 26, Type III; *n* = 0) were included in the FAI group. After the introduction of AIIS decompressions at the primary author’s institution in April 2013, arthroscopic FAI correction and labral preservation with AIIS decompression was performed if patients had symptomatic AIIS impingement/deformity. Twenty-nine patients who underwent arthroscopic FAI surgery with AIIS decompression were included in AIIS group (Type I; *n* = 5, Type II; *n* = 24, Type III; *n* = 0). The decision to perform AIIS decompression was based on a physical examination, including terminal pain at limited straight flexion [14], tenderness to palpation over the AIIS that re-created the terminal flexion discomfort, a positive subspinal impingement test, which consisted of passive hip flexion in neutral rotation [28], 3D-CT imaging revealing abnormal AIIS morphology (i.e., type II or III) and arthroscopic findings showing focal bruising and synovitis at the level of the AIIS. For cases of symptomatic subspinal-related impingement (clinical and intraoperative confirmation) in the presence of type I AIIS deformity on 3D-CT imaging, AIIS decompressions were also performed. There was no significant difference for AIIS type/classification between groups (n.s.).

### Surgical technique

Supine hip arthroscopy was performed with a well-padded perineal post on a traction table under general and epidural anesthesia. An anterolateral portal (ALP), a mid-anterior portal (MAP) and a proximal mid-anterior portal (PMAP) were established. An interportal capsular cut using a Beaver knife (Becton Dickinson, Franklin Lakes, NJ) was performed to improve accessibility of the arthroscope and the surgical instruments. Labral repair with suture anchors was performed whenever possible, and labral reconstruction was performed with an iliotibial band autograft harvested from the ipsilateral side if the labrum was irreparable. Microfracture was performed if cartilage damage (grade III and IV) was observed.

Additionally, AIIS decompression was performed proximally for routinely 1–1.5 cm using a motorized round-headed burr through the MAP in all of the AIIS groups. Occasionally, in cases in which further decompression was required, the burr had limited accessibility to the AIIS. A small capsular/rectus window was then created with a radiofrequency probe through the capsule and rectus tendon. Further AIIS decompression was done [12].

After releasing traction, arthroscopic femoroplasty was performed with dynamic confirmation of impingement-free range of motion. Finally, capsular closure was performed to stabilize the joint as previously described [33].

### Rehabilitation protocol

All patients in both groups underwent the same postoperative rehabilitation protocol. Our postoperative rehabilitation protocol comprises four phases (Phase 1: protection period, protection of the repair site; Phase 2: interim period, acquisition of normal walking; Phase 3: advanced period, muscle and muscle endurance recovery; Phase 4: athletic rehabilitation period, return to sports). In Phases 1 and 2, patients were placed in a hip brace and instructed to be flat-foot weight bearing on the operated leg for the first 2 weeks. Gentle passive ROM exercises were initiated during the first week under the supervision of a physical therapist. Circumduction was carried out at 70° of hip flexion and neutral hip flexion for the first 2 weeks. Continuous passive motion exercises were used to avoid adhesive capsulitis by applying 0°–90° of hip flexion for up to 2 h/day for 2 weeks. In phase 3, endurance strengthening was commenced only after ROM was maximized and after good stability in gait and movement was demonstrated. Patients were allowed to progress their physical activity only after passive ROM was symmetric and pain free, and they demonstrated a normal gait pattern [22].

### Assessment

Both groups were matched, and there was no significant difference between the AIIS group and FAI group with regards to AIIS morphology, sex, mean age, body mass index (BMI), labral repair/reconstruction, or microfracture. (Table 1) Patient-reported outcome (PRO) scores were obtained using the modified Harris hip score (MHHS) [2], nonarthritic Hip Score (NAHS) [6] and iHOT-12 [11] preoperatively and 6 months after surgery.

**Table 1:** Univariate analysis comparing demographic data for AIIS and FAI groups

	AIIS group (*n* = 29)	FAI group (*n* = 31)	*p* value
Sex	Male: 14 Female: 15	Male: 20 Female: 11	n.s.
Age, years	27.5 ± 12.0	31.6 ± 13.5	n.s.
Body mass index, kg/m^2^	22.5 ± 3.2	21.4 ± 2.1	n.s.
Labral repair/reconstruction	Repair: 28 Reconstruction: 1	Repair: 29 Reconstruction: 2	n.s.
Micro fracture	Not done: 28 Done: 1	Not done: 31 Done: 0	n.s.
AIIS type	Type I: 5 Type II: 24	Type I:5 Type II: 26	n.s.

There were no significant differences for gender, age, body mass index, labrum treatment, microfracture rate, and AIIS type between groups

Data are presented as the mean unless otherwise indicated. Student’s *t* test and *χ*^2^ test

### Muscle strength assessment

Knee extensor and hip flexor muscle strength in both involved and non-involved sides, preoperatively and at 6 months after surgery, was evaluated by the same experienced tester. The strength of muscle examination was performed using a hand-held dynamometer (HHD) (Power Track 2 TM COMMANDER, Japan, Medix). It has also been performed by fixing the lower leg and femur at a distal site to increase the reproducibility of the examination [16]. The strength of knee extensor and hip flexor were measured with the knee at 90° flexion in a sitting position [1]. No accessory movements of other body parts other than the tested limb were allowed. To familiarize the patients with the test, they were allowed to practice before the data acquisition trials. The maximum muscle strength was assessed in 3 trials with 30 s rest between trials. The weight ratio (N/kg) was calculated from the highest value of the 3 trials’ isometric maximum voluntary contraction (MVC) to standardize the strength difference due to physique variability. All muscle strength data were normalized to body weight according to the following formula: (N strength/kg subject’s body mass). A dynamometer was used to calculate the coefficient variation of the measured value in this study. It adopted the subsequent data with less than 15% coefficient variation to suppress the dispersion of the data. When the coefficient variation was 16% or more, we re-measured to obtain reliable data. The reliability of muscle strength measurement was examined beforehand for healthy subjects. The test–retest reliability was determined using the intraclass correlation coefficient (ICC). The intra-rater reliability for 20 lower extremities of 10 healthy subjects was investigated (ICC(1, 1): 0.95–0.97). The interrater reliability for 18 lower extremities of 9 healthy subjects was investigated (ICC(2, 1): 0.89–0.93).

Approval for the study was granted through the institutional review board IRB (University of Occupational and Environmental Health, approval number: H28-097).

### Statistical analysis

In the AIIS group, bilateral knee extensor MVC strength before and 6 months after surgery was evaluated using a paired *t* test. For both the AIIS and the FAI group, the hip flexor MVC strength in the involved side preoperatively and 6 months after surgery was analyzed using a paired *t* test.  For both the AIIS and the FAI group, involved side and non-involved side hip flexors MVC strength preoperatively and 6 months after surgery was evaluated using a paired *t* test.

The Wilcoxon rank sum test and Mann–Whitney *U* test were performed to compare clinical scores between groups. The difference of the revision surgery rate between groups was evaluated using Fisher’s exact test. Statistical analyses were performed using Stat Flex software (ver. 6), and the level of significance was set at *p* < 0.05.

A priori power analysis was performed using G* power 3 under the following conditions (paired *t* test, power 0.8, significance level *p* = 0.05) [3]. The results of this analysis were effect size *d* = 0.75, total sample size = 17, and actual power = 0.81.

## Results

There was no significant difference for preoperative knee extensor strength between the involved side and non-involved side for the AIIS group (n.s.) (Table 2). In the AIIS group, there was no significant difference between preoperative and postoperative knee extensor strength for the involved side (n.s.) (Table 2). There was also no difference for postoperative knee extensor strength between the involved side and non-involved side (n.s.) (Table 2).

**Table 2:** The strength of knee flexor and hip flexor in AIIS and FAI group

	Pre-op.		Post-op.	
	Involved side	Non-involved side	Involved side	Non-involved side
AIIS group (*N* = 29)				
Knee extensor strength (N/kg)	4.2 ± 1.9	4.2 ± 1.9	4.3 ± 1.2	4.3 ± 1.3
Hip flexor strength (N/kg)	2.5 ± 1.0*	2.8 ± 0.9	2.9 ± 1.4^†,§^	3.2 ± 1.5
FAI group (*N* = 31)				
Hip flexor strength (N/kg)	2.9 ± 1.0**	3.2 ± 1.1	3.2 ± 1.3^‡^	3.4 ± 1.4

Data represent mean ± SD

*p < 0.05 compared with non-involved side in pre-op.

**p < 0.01 compared with non-involved side in pre-op.

^†^*p* < 0.05 compared with non-involved side in post-op.

^‡^*p* < 0.01 compared with non-involved side in post-op.

^§^*p* < 0.05 compared with involved side in pre-op.

Pre- and postoperative hip flexor strength was examined for both AIIS and FAI groups to evaluate the effects of AIIS decompression on hip flexor strength. For both the AIIS and FAI groups, preoperative hip flexor strength for the involved side was significantly decreased compared to the non-involved side (AIIS group: *p* < 0.05, FAI group: *p* < 0.01). Only the AIIS group had improved postoperative hip flexor strength compared to preoperative measures (*p* < 0.05) (Table 2). However, in both AIIS and FAI groups, postoperative hip flexor strength for the involved side remained significantly decreased compared to the non-involved side (AIIS group: *p* < 0.05, FAI group: *p* < 0.01) (Table 2).

Pre- and postoperative MHHS, NAHS and iHOT-12 were examined between groups (Table 3). There was no significant difference between groups for preoperative MHHS (n.s.). For both the AIIS and FAI groups, the mean MHHS significantly improved postoperatively (AIIS group: *p* < 0.001, FAI group: *p* < 0.001). Postoperatively, there were no significant differences in MHHS between AIIS and FAI groups (n.s.). There were no significant differences regarding preoperative NAHS between both groups (n.s.). For both the AIIS and FAI groups, the mean NAHS significantly improved postoperatively (AIIS group: *p* < 0.001, FAI group: *p* < 0.001). There were no significant differences for postoperative NAHS between both groups (n.s.).

**Table 3:** MHHS, modified Harris Hip Score

	AIIS group (*N* = 29)		FAI group (*N* = 31)	
	Pre-op.	Post-op.	Pre-op.	Post-op.
Modified Harris Hip Score	75.7 ± 13.8 (46.2–96.8)	93.7 ± 7.2^**^ (72.6–100)	72.5 ± 14.8 (38.5–95.7)	89.6 ± 15.2^**^ (47.3–100)
Nonarthritis Hip Score	57.3 ± 11.6 (35.0–80.0)	71.7 ± 7.1^**^ (54.0–80.0)	54.3 ± 13.5 (30.0–78.0)	66.3 ± 13.1^**^ (30.0–80.0)
i HOT-12	48.4 ± 20.5 (9.1–83.8)	82.8 ± 12.8^**,*^ (53.5–100)	44.7 ± 18.0 (18.1–83.0)	66.3 ± 22.4^**^ (14.1–100)
Revision rate	0%(0/29)^†^		19.4% (6/31)	

NAHS, nonarthritis hip score in AIIS and FAI group. iHOT-12. in AIIS and FAI group. The number of revision surgery in AIIS and FAI group

The mean postoperative iHOT-12 in AIIS group was significantly higher than that in FAI group

Data represent mean ± SD (range)

*p < 0.01 compared with FAI group in post-op.

^**^p < 0.001 compared with pre-op.

^†^*p* < 0.05 compared with FAI group

There were no significant differences regarding preoperative iHOT-12 between both groups (n.s.). For both the AIIS and FAI groups, the mean iHOT-12 significantly improved postoperatively (AIIS group: *p* < 0.001, FAI group: *p* < 0.001). Moreover, the mean postoperative iHOT-12 in the AIIS group was significantly higher than that in the FAI group (*p* < 0.01) (Table 3).

**Fig. 2:**
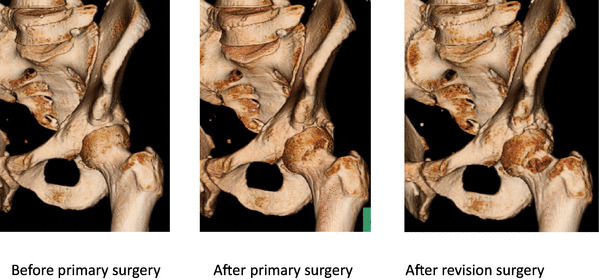
Images of 3D-CT before the primary operation, postoperatively, after revision surgery. It shows that the bulge of AIIS remaining after primary operation has disappeared after revision surgery

Finally, the revision surgery rates were analyzed between group differences. The revision surgery rate for the AIIS group was significantly lower compared with that in the FAI group (*p* < 0.05) (Table 3). For the FAI group, the reasons for revision surgery were 5 residual symptomatic AIIS deformities (all cases were type II), residual cam deformities in four hips, residual pincer deformity in one hip, and capsular adhesions in two hips. There were no cases of conversion to THA. Revision surgery was performed at a mean of 16 ± 10 months (range 6–29) postoperatively. 3D-CT imaging revealed residual deformities requiring revision arthroscopic hip surgery and corrections after the revision procedures (Fig. 2).

## Discussion

The primary findings of the current study were as follows: (1) there were no knee extensor strength differences pre- and postoperatively for the AIIS group; (2) AIIS decompression resulted in significantly improved hip flexor strength at 6 months postoperatively compared to preoperative measures; and (3) the mean postoperative iHOT-12 in the FAI group was significantly lower than that in the AIIS group. In addition, the revision rate in the AIIS group was significantly lower than that in the FAI group and was largely due to persistent AIIS deformity.

Muscle strength measurement was performed by HHD in this study. The previous 19 studies that compared HHD to isokinetic testing were reported to have a moderate-to-good reliability and validity when compared with isokinetic testing [32]. Muscle strength data with small coefficient variation was used to improve reliability of muscle strength measurement. It has been clarified in advance that the reliability of the muscle strength measurement method in this study is high. Therefore, HHD is an effective tool for muscle strength measurement.

Knee extensor and hip flexor strength were measured with the knee at 90° flexion in a sitting position. Markhede has demonstrated that the rectus femoris was responsible for approximately 40% of hip flexion torque at 90° hip flexion [21]. In addition, Shenoy et al. reported that the ratio of the rectus femoris to quadriceps femoris activation on an electromyogram was approximately 30% with active extension of the knee starting at 90° flexion [31]. From the abovementioned evidence, our findings indicate that muscle function of the rectus femoris can be reflected by the muscle strength values of both the knee extensor and hip flexor.

There is a study displaying hip muscle weakness (isometric and isokinetic) in symptomatic patients with FAI compared to a control population [4]. However, there is no report regarding knee extensor muscle weakness in patients with FAI. In this study, there were no significant preoperative knee extensor strength differences between the involved and non-involved sides. These findings suggest that arthroscopic FAI correction does not affect knee extensor muscle strength. AIIS avulsions, which include the origin of the rectus femoris, have been reported to result in hip flexor and knee extensor strength deficits [10]. A recent study has shown that hip flexor muscle strength is decreased after arthroscopic FAI surgery [3]. If an overly aggressive resection of the AIIS is performed, the direct head of the rectus femoris could potentially be partially or completely detached from its origin (AIIS), and this could result in potential hip flexor and knee extensor strength deficits.

There are several studies reporting clinical outcomes after AIIS decompression. Hapa et al., in a clinical and cadaveric study, reported that arthroscopic AIIS decompression as a part of a FAI corrective procedure can be performed without significant rectus femoris compromise by decompressing 1–1.5 cm from the intracapsular side. They reported the largest case series (163 cases) with subspine decompression and demonstrated improved clinical outcomes in the patients with FAI combined with AIIS impingement [12]. In contrast, Nwanchukwu et al. reported a series of 33 patients with AIIS/subspinal impingement where arthroscopic AIIS decompression was performed without concurrent treatment of FAI [25]. They reported improved outcomes; however, 3 of 33 patients required revision arthroscopic surgery. AIIS/subspine impingement frequently occurs between the AIIS and anteromedial femoral neck/head–neck junction. Therefore, when a cam deformity or decreased head–neck offset is present along with a prominent AIIS, the current authors believe decompression of both entities is appropriate. Further research comparing AIIS decompressions with and without cam resections would be required to better define the role for isolated AIIS deformity in the presence of mild cam-type impingement. AIIS decompression was performed utilizing the same surgical technique attempting to minimize subspinal decompression to approximately 1–1.5 cm above the acetabular rim. In this study, our findings indicate that the AIIS decompression technique is capable of resecting only the inferior edge of the AIIS without invading the rectus femoris and did not alter knee extensor strength postoperatively compared to preoperative measures.

Casartelli et al. also demonstrated isokinetic hip flexor muscle weakness in symptomatic FAI patients compared to a control population [3]. Our results were similar; however, we compared the isometric muscle strength of the involved side to the non-involved side of the same individual. Another study demonstrated that hip flexion weakness remains up to 2.5 years after the FAI surgery [3]. We usually set the goal for return to full play after surgery at 4–6 months after surgery. However, there are no reports regarding the extent of recovery of hip flexor strength postoperatively. In this study, for both AIIS and FAI groups, the strength of the hip flexor in the involved side remained significantly decreased compared to the non-involved side at 6 months after surgery. However, in the AIIS group, the strength of the hip flexor was significantly improved from preoperative measures in contrast to the FAI group. These results indicate that the hip flexor strength after arthroscopic AIIS decompression as a part of FAI correction might recover more quickly compared to that after arthroscopic FAI correction without AIIS decompression secondary to improved impingement-free flexion range of motion. The current study reveals that AIIS decompression as part of a FAI corrective procedure does not appear to alter hip flexor strength postoperatively. More notably, hip flexor strength improved in the AIIS decompression group and might be secondary to less pain with hip flexion testing. Hip flexion testing in the presence of AIIS impingement might create tension at the level of the AIIS and anterior hip capsule and labrum and account for this difference between groups. This discrepancy, therefore, might be the result of pain inhibition of the hip flexors in the setting of AIIS impingement. AIIS decompression, when clinically indicated, appears be beneficial with regard to hip flexor strength. AIIS decompression for FAI correction improved postoperative PRO scores without altering the muscle strength of the hip flexor and knee extensor.

It was also revealed that iHOT-12 in the AIIS group improved significantly compared with the FAI group. The mean postoperative iHOT-12 in the AIIS group was significantly higher than that in the FAI group, while there were no significant differences of postoperative MHHS and NAHS between both groups. A recent study has shown that MHHS has a significant ceiling effect, limiting its utility as an outcome measure in active patients [17]. NAHS comprises 10 questions from WOMAC and is potentially undermined by ceiling and floor effects [5]. In fact, iHOT-12 has been developed and shortened to iHOT-33 to provide excellent evaluation for managing nonarthritis hip problems in young active patients. It also has excellent psychometric properties [11]. The PRO used in this study includes items of ADL that require a large hip flexion angle. In cases where AIIS impingement remained in the FAI group, there was a possibility that there were difficulties with ADLs requiring greater hip joint flexion. Postoperative iHOT-12 scores were significantly higher in the AIIS group compared to the FAI group. However, there were no between-group differences regarding postoperative MHHS and NAHS, which may have been due to the ceiling and floor effect of those respective outcome measures [5, 17].

Moreover, the revision rate in the FAI group is significantly higher than that in the AIIS group. Although the reasons for revision were multifactorial, 5 of 6 patients requiring revision had residual AIIS deformities. These findings indicate that an AIIS decompression is one of the most important key points in the presence of a symptomatic AIIS deformity in order to optimize outcomes and minimize failure rates.

There are certain limitations for this study that should be mentioned. This is a retrospective study with all the inherent limitations of such a study design. There were many cases in which rehabilitation follow-up was limited in our hospital because many surgery cases have come over from all over the country. Selection bias exists in our study because a number of patients were excluded, as they were seen at clinic sites where dynamometer testing was not performed. In addition, the sample size was relatively small and the follow-up was short term. Further studies are needed to evaluate the longer-term effects of various surgical procedures on hip function and muscle strength in a larger number of patients. It was unclear as to whether the addition of an AIIS decompression had an effect on the ultimate outcome other than the muscle strength measures noted previously. Postoperative inflammation of the origin of the direct head of the rectus femoris may theoretically exist and affect patient function as well. Imaging studies, such as magnetic resonance imaging, may be necessary to examine the effect of AIIS decompression on the origin of the rectus femoris. Finally, it is difficult to distinguish whether the most important cause of revision was due to AIIS impingement or residual FAI.

## Conclusion

Arthroscopic management of AIIS impingement based on clinical and or radiographic findings results in improved clinical outcomes, lower revision rates, and has no adverse effect on hip flexor/knee extensor muscle strength.
